# A feasibility study to investigate the utility of a home-based exercise intervention during and after neo-adjuvant chemotherapy for oesophago-gastric cancer—the ChemoFit study protocol

**DOI:** 10.1186/s40814-020-00597-y

**Published:** 2020-04-23

**Authors:** J. Chmelo, A. W. Phillips, A. Greystoke, S. J. Charman, L. Avery, K. Hallsworth, J. Welford, R. C. F. Sinclair

**Affiliations:** 1 Northern Oesophago-gastric unit, Royal Victoria Infirmary, Newcastle upon Tyne Hospitals NHS Foundation Trust, Newcastle upon Tyne, UK; 2 Translational and Clinical Research Institute, Faculty of Medical Sciences, Newcastle University, Newcastle upon Tyne, UK; 3 School of Medical Education, Newcastle University, Newcastle upon Tyne, UK; 4 Northern Centre for Cancer Care, Freeman Hospital, Newcastle upon Tyne Hospitals NHS Foundation Trust, Newcastle upon Tyne, UK; 5 Cardiovascular Research, Translational and Clinical Research Institute, Faculty of Medical Sciences, Newcastle University, Newcastle upon Tyne, United Kingdom; 6 Newcastle upon Tyne Hospitals NHS Foundation Trust, Newcastle upon Tyne, UK; 7 Centre for Rehabilitation, Exercise and Sport Science, School of Health & Life Sciences, Teesside University, Tees Valley, UK; 8 The Liver Unit, Newcastle upon Tyne Hospitals NHS Foundation Trust, Newcastle upon Tyne, UK; 9 Hepatology Research, Translational and Clinical Research Institute, Faculty of Medical Sciences, Newcastle University, Newcastle upon Tyne, UK; 10 Department of Anaesthesia and Critical Care, Royal Victoria Infirmary, Newcastle upon Tyne Hospitals NHS Foundation Trust, Newcastle upon Tyne, UK

**Keywords:** Prehabilitation, Oesophageal cancer, Gastric cancer, Home-based exercise, Neo-adjuvant chemotherapy

## Abstract

**Background:**

Treatment of locally advanced oesophago-gastric adenocarcinoma usually entails neo-adjuvant chemotherapy (NAC) and surgery. Surgery is associated with high morbidity and mortality. Cardiopulmonary reserve of patients having major surgery is related to postoperative outcomes. Complications are associated with poorer quality of life and may affect prognosis. Preventing complications may be beneficial to both of these and have cost implications. Prehabilitation may improve recovery from surgery by increasing a patients’ fitness before surgery. Designing a potentially cost and resource effective regimen which improves cardiopulmonary reserve may have a beneficial impact on patient outcomes after surgery.

**Methods:**

The ChemoFit study is a non-randomised, single-arm and single-centre pilot study designed to investigate the feasibility of a home-based prehabilitation exercise intervention for patients receiving neoadjuvant treatment prior to oesophago-gastric surgery. Forty patients will be recruited at a single high-volume centre. The simple, home-based exercise intervention involves patients increasing their daily step-count during and after NAC and in the weeks leading up to surgical resection of the cancer. Additionally, quality of life assessments (QLQ-C30 and QLQ-OG25), oncological treatment delivery and participant perceptions of the study assessed by focus groups and questionnaires will be performed. The primary outcomes are to assess feasibility of the exercise intervention. The secondary outcomes will evaluate changes in cardiopulmonary reserve, sarcopenia and fat composition.

**Discussion:**

It is anticipated that during an important teachable moment, the diagnosis and treatment of cancer, our patients will be open to the possibility of improving their fitness during chemotherapy and before major cancer surgery. It is possible that the negative impact of NAC on cardiopulmonary fitness could be prevented by implementing a home-based prehabilitation programme during and after NAC, prior to surgery for oesophago-gastric adenocarcinoma.

**Trial registration:**

This study has been approved by the Health Research Authority (REC 18/WA/0427). Newcastle upon Tyne Hospitals NHS Foundation Trust (NUTH) will act as the study sponsor and the work is funded by a grant awarded by The Jon Moulton Charitable Foundation, supported by a research post funded by the Sir Bobby Robson Foundation. Trial registration: Clinicaltrials.gov, NCT04194463. Registered 11th December 2019—retrospectively registered, https://clinicaltrials.gov/ct2/show/NCT04194463.

## Introduction

Curative treatment of locally advanced oesophago-gastric adenocarcinoma usually entails neo-adjuvant chemotherapy (NAC) followed by surgery and then postoperative adjuvant chemotherapy. Multimodal therapy has been shown to be superior to surgery alone [1, 2]. This type of major surgery is unfortunately associated with high morbidity and mortality [3, 4]. It has been shown that cardiopulmonary fitness prior to major surgery is related to postoperative outcomes [5]. Previous work has demonstrated a significant and sustained reduction in fitness during NAC given for oesophago-gastric cancer, which not only affects daily activities and quality of life, but also decreases the physical reserve of the patient before they undergo major surgery [6, 7]. Both a reduction in objective cardiopulmonary reserve, measured by cardiopulmonary exercise testing (CPET), and reduction in muscle mass (sarcopenia) on CT scan analysis in the interval between commencing chemotherapy and undergoing surgery has previously been observed [7]. Complications of major surgery are associated with poorer quality of life in the short-term and worse prognosis [8]. Further, they can delay or prevent the administration of post-operative chemotherapy, which may impact on disease recurrence. Preventing these complications may improve surgical outcomes, patient health and survival from oesophago-gastric cancer. In addition, exercise may have beneficial effects on improving immune surveillance and reducing inflammation; reversing abnormalities that may be associated with tumour recurrence and growth [9].

High-intensity exercise interventions have already demonstrated the ability to prevent the decline in health observed in other patient groups undergoing chemotherapy [10, 11]. These programmes require a large financial investment, time commitment and the use of complex hospital resources. In a wide geographical area, attending hospital-based interventions can be challenging to implement, particularly when patients are unable to travel due to the impact of their treatment, and the cost of travelling long distances. To help overcome these barriers, we have developed a home-based exercise intervention, which could be utilised by a large number of patients at any one time. The aim of the pilot study is to assess the feasibility, acceptability and safety of this intervention on a small group of patients. Further, the impact of such a regimen on cardiorespiratory fitness and sarcopenia will be assessed and its effect on biomarkers measuring immune function and frailty explored. It is hoped that the data from this study will help to inform future randomised studies into the benefit of exercise programmes.

## Methods

### Study design

This is a prospective, single-group, single-centre pilot study investigating the feasibility and acceptability of a home-based exercise intervention during oncological treatment of patients presenting with operable advanced adenocarcinoma of oesophagus, gastro-oesophageal junction and stomach. Patients will be invited to participate in the study which involves provision of a home-based exercise intervention before, during and after NAC, and in the weeks leading up to surgical resection of the cancer.

### Study aims

The primary aim of the pilot study is to assess the feasibility of the home-based exercise intervention. This will be assessed by the measurement of the recruitment rate, completion rate and individual compliance with the intervention (i.e. do patients perform the exercise, engage with telephone support and record their activity levels in accordance with protocol).

The secondary aim of this study involves the preliminary assessment of benefit to patients’ physiology and quality of life related to the exercise intervention. Results will be used to inform the design of a larger scale randomised controlled trial evaluating the impact of an exercise regimen on patients’ cardiopulmonary fitness, sarcopenia, quality of life and potentially postoperative outcomes such as morbidity and length of hospital stay. The ChemoFit study (as a part of the secondary outcomes) will aim to assess cardiopulmonary fitness as measured using cardiopulmonary exercise testing (CPET), changes in muscle mass (sarcopenia) as measured using computed tomography (CT), handgrip strength and quality of life utilising QLQ-C30 and QLQ-OG25 questionnaires [12, 13]. Patient’s physical activity will be measured by daily step counts using waist-worn pedometers, which serve as the method of quantifying activity levels and will help with prescribing home-based activity increases. The oncology treatment will be assessed by recording chemotherapy dose reduction and delays, percentage of planned chemotherapy delivered and admissions to hospital with chemotherapy induced toxicity. Acceptability of the intervention will be assessed with patients taking part in the study by eliciting their views and experiences via focus group discussions. The qualitative component of this feasibility study will obtain participants’ views and experiences of the intervention via focus groups, including what they perceive to be barriers and facilitators to using it in order to assess acceptability. The purpose of this qualitative component is to inform the design of future studies and there is no plan to include acceptability or any other qualitative work in future designs. Up to five focus group discussions will be conducted by a researcher using a topic guide containing questions designed to address the objectives of the study.

It is not intended to use any data obtained from the ChemoFit study in future randomised controlled trials.

### Inclusion and exclusion criteria

Patients with locally advanced adenocarcinoma (T3+, T1/2 N+) [14] of the oesophagus, oesophago-gastric junction or stomach and a planned NAC regimen including the locally used ECX, ECX variant [1] or FLOT [15] regimens will be eligible to participate in the study. Participants 18 years of age or older and able to consent to the study and carry out the planned intervention and CPET tests required in the protocol will be invited to participate.

Exclusion criteria are patients treated with neo-adjuvant radiotherapy or chemoradiotherapy, patients in whom CPET is contraindicated [16], and patients with inoperable tumours.

### Patient selection and consent process

Patients will be identified during the multidisciplinary cancer staging process at the Northern Oesophago-gastric Unit. Suitable patients, who meet the eligibility criteria, will be provided with a patient information sheet (PIS) explaining the study. They will be consented for the study after the finalised oncological treatment plan has been agreed by the multidisciplinary team and discussed with the patient. By providing a PIS early in the staging process patients will have sufficient time to consider their participation in the study and ask questions. All eligible patients will be approached, and participation will be voluntary: they will be able to withdraw consent at any point during the study. Patients who have not agreed to take part in the study by the time they start their first chemotherapy session will not be eligible to participate.

During the first study enrolment meeting, informed, written consent will be obtained prior to any study procedures and each patient will be asked to complete baseline QLQ-C30 and QLQ-OG25 quality of life questionnaires [12, 13]. The handgrip strength of their dominant hand will be measured using grip strength dynamometer (T.K.K. 5101 GRIP-D, Takei scientific instruments Co., Ltd., Japan). Participants will be provided with a simple, easy to use pedometer (Walking style One 2.1, Omron Healthcare UK Ltd., UK), resistance band (BodyMax resistance tube, BodyMax Ltd., UK) and exercise diaries. They will receive information about what the exercise intervention involves and taught how to use the pedometer, resistance band and exercise diaries.

### Intervention

#### Baseline measurement

**Fig. 1:**
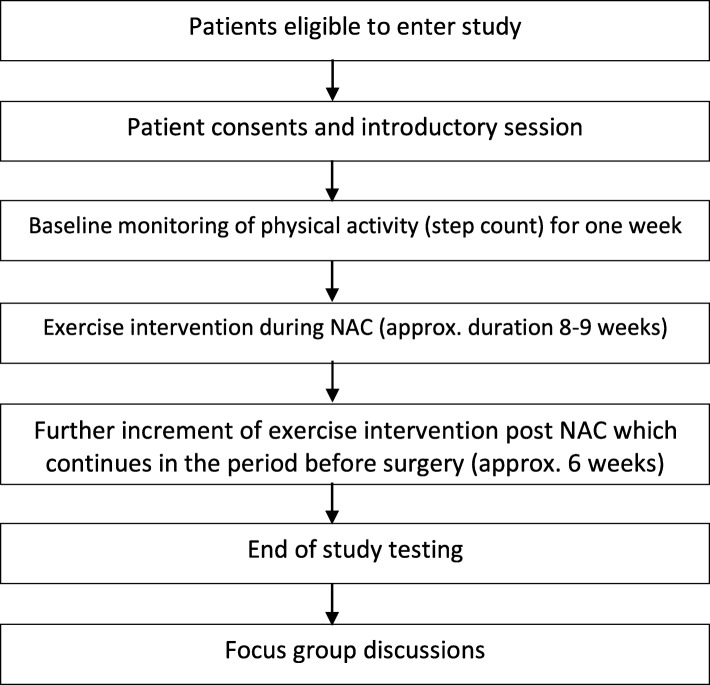
Consort diagram of the study

Once enrolled into the study, participants will immediately enter a 1-week period of monitoring using a pedometer to record their habitual physical activity (daily step count). This initial period will be used to establish a baseline physical activity level for each participant and will be used to calculate each individual’s median daily step count. Baseline CPET and CT data will be collated at this point. Both CPET and CT data will have been performed as part of routine clinical care during the multidisciplinary team (MDT) staging process (Fig. 1).

#### The exercise intervention

The exercise intervention will start immediately following completion of the 1-week period of baseline monitoring. The intervention will continue during chemotherapy and afterwards in the time period between the end of chemotherapy and the planned surgical resection. The intervention will be personalised to each patient based on their baseline level of activity (as recorded by a pedometer during the first week of observation), age, general health, motivation, and social circumstances with the aim of achieving the greatest improvement in their cardiopulmonary fitness.

The exercise intervention will aim to achieve an increase in daily step count of 2000 steps per day, 7 days per week [17]. This increase in physical activity (step count) will be achieved by walking or jogging at moderate intensity for a target of 30 min per day, each day (this is one ‘bout‘ of moderate intensity exercise) [18]. Participants will be instructed on how to achieve moderate intensity physical activity using the modified Borg rating of perceived exertion (RPE) scale (0–10) aiming to achieve an RPE level between 3 and 4 (moderate to somewhat strong exertion) [19]. This will ensure that the intensity at which these 30-minute bouts of physical activity are maintained throughout the entire exercise period. Patients will be asked to record their daily step count at the end of every day and the RPE for the 30-min bout of exercise. The Borg scale will be used as a tool to describe levels of home-based physical activity throughout the study.

After the first week of the intervention, patients will be contacted by a member of the research team; and with the research team’s support they will be given the option to maintain or to increase their step count further if they feel that they have managed to achieve the prescribed amount of steps in the previous week. The same approach will be used after each week of the intervention. The aim of this is to facilitate a participant-led increase in daily physical activity based upon increasing the daily step target. Participants will also be encouraged to perform other physical activities like jogging, swimming, cycling or group activities if they wish and feel able to do so.

The participants’ progress will be monitored in a daily diary entry.

After completion of NAC, a further enhanced increase in the daily activity above the current physical activity level will be implemented providing the patient is comfortable with this progression. The level of this increase will be based on current progress and, as such will be tailored to individual needs and circumstances. This incremental period will start 7 days after the last Capecitabine tablet (given as part of the chemotherapy) and continue until the end of the study and the last study visit.

#### Strengthening exercises

Strengthening exercises will be performed alongside the exercise intervention. Strengthening exercises will be performed every day, 7 days per week, and will start after the end of the baseline observation week has been completed. The patients will be taught how to perform two sets of five simple exercises (sit to stand/wall squat, biceps curl, upright row, leg abduction, wall press), each for 1-min duration by a member of the research team. This equates to 10 min of strengthening exercise in total each day. Exercises have been selected to strengthen major body muscle groups. Each exercise will be prescribed at two levels of intensity in order to tailor them to individual fitness levels. A booklet demonstrating the correct performance of the strengthening exercises will be given to each patient to take home. They will receive weekly telephone calls to encourage and reinforce the importance and conduct of these strengthening exercises.

#### Interim analysis of step-based regimen

Once the first 10 participants have each completed the first week of the exercise regimen during NAC, we will perform an interim analysis of their achieved daily step count during this first week. If ≥ 60% of participants have not achieved the prescribed daily step count for at least 5 days of this week, then we will lower the increment in steps described within the protocol. We will change this initial increment to 1000 steps above baseline daily step count for all participants recruited after the interim analysis.

Conversely, if ≥ 80% of participants have achieved the prescribed daily step count for all 7 days of the first NAC exercise week, we will increase our first increment to 3000 steps above baseline daily step count for all participants recruited after the interim analysis.

This strategy has been chosen to ‘fine adjust’ our initial step increment after the first observation week as it is difficult to predict how demanding this increment is going to be for patients, especially in the context of starting chemotherapy treatment. There is no current evidence to guide this decision. Based on the findings from the Active-at-Home HF pilot study in stable chronic heart failure patients, we replicated the same physical activity steps/day in our patients, i.e. an increase of 2000 steps/day from baseline physical activity [17].

#### Weekly telephone reporting, reinforcement and encouragement

A member of the research team will contact each study participant once per week by telephone in order to provide support, reinforce the aims of programme and reiterate the benefits, monitor activities and exercises, collect the previous week’s data and provide positive feedback. Participants will be encouraged to reflect upon the previous weeks achievements and discuss any problems or factors which may have inhibited progress with a view to identifying strategies to overcome these problems, where possible. These will be recorded by the research team. Patients will be given the opportunity to increase their daily target for the next week if they feel that this is achievable or appropriate.

#### Completion of the exercise intervention

The end of the study period is defined as the last full week of exercise before surgery. Participants will complete an end of study CPET, hand grip strength measurement, blood sampling and quality of life questionnaires during the 2 weeks prior to their scheduled surgery. A CT scan, which is performed as part of routine clinical care prior to the surgery, will be used for comparison with the initial CT scan to measure sarcopenia.

#### Cardiopulmonary exercise testing

The end of study CPET will be performed in accordance with the American Thoracic Society/American College of Chest Physicians guidelines [20]. Patients will perform a symptom-limited continuous ramped test on cycle ergometer (Ergoselect 200, Ergoline GmbH, Germany). Metabolic gas analysis will be conducted via the metabolic cart (Ultima Series, MGC Diagnostics, USA). CPET data will be analysed using the Breeze SuiteTM software (Ultima Series, MGC Diagnostics, USA). This will be performed initially alongside staging investigations, which is part of normal routine clinical care and additionally prior to surgery as part of the study protocol.

#### Usual clinical care during the study

All participants enrolled in the study will be managed according to the standardised patient pathway that already exists. Patients are reviewed by the surgical team and counselled with regard to the treatment options that are available. Those patients that are struggling nutritionally are then reviewed by one of two specialist upper gastrointestinal dietitians. If patients are unable to manage sufficient calorie input without assistance, they may have a naso-enteric feeding tube placed or a feeding jejunostomy so that they receive sufficient calories.

### Study outcomes

The primary and secondary outcomes are defined in Table 1 and Table 2.

**Table 1:** The primary outcomes of the study

Primary outcome measures	Definition	Success threshold
Recruitment rate	Proportion of all patients approached who meet the eligibility criteria and agree to enter the study	*50%*
Completion rate	Proportion of all patients that enter the study that remain participants at the end of the defined study period	*75%*
Compliance	Percentage of intervention days when the patient was wearing his/her pedometer, was contactable every week and was recording his/her daily step count	*75%*

**Table 2:** The secondary outcomes of the study

Secondary outcome measures	
CPET	Change in VO2 peak (ml/min/kg), change in VO2 at AT (ml/min/kg) and change in FEV1 prior and post intervention
Sarcopenia	Change in amount of L3 level skeletal muscle area determined on CT scan by methodology described by Perthen et al. [21], change in fat composition and volume measured by CT scan and change in grip strength pre and post intervention
Exercise intervention	Change of step count throughout the intervention
Oncological treatment	Number of patients with dose reductions or who terminate chemotherapy early, percentage of planned chemotherapy delivered, admissions to hospital with chemotherapy induced toxicity
Quality of life	Change of score on validated quality of life questionnaires QLQ-C30 and QLQ-OG25 pre and post intervention
Acceptability	Qualitative evidence generated from focus group discussions with participants

### Statistical considerations

#### Estimation of sample size

A sample size of 40 participants has been selected in accordance with published guidance for pilot feasibility studies [22, 23]. Further, this was considered to be an achievable number of patients to recruit within the 12 months. Approximately 130 patients undergo oesophago-gastric resections each year for cancer at the Northern Oesophago-Gastric Unit, Royal Victoria Infirmary, Newcastle upon Tyne. It is estimated that 70% will be eligible to take part in the study (i.e. will receive preoperative chemotherapy for adenocarcinoma) [24]. All of these patients will be invited to participate, and it is hoped that enrolment will reach approximately 85–95% as already demonstrated by other prehabilitation studies, which employed home-based interventions [25–27]. In this respect, a sample size of 40 also realistically represents the number of patients that might be expected to be recruited in a year. In a recent study investigating CPET testing after chemotherapy only one participant of 32 recruited participants withdrew consent during the study (3%) [24]: this group of cancer patients is motivated and compliant. It is expected that it will take 12 months to recruit 40 patients to this study.

#### Statistical analysis

Analyses will be conducted using SPSS, version 24.0 (IBM Corp., USA). The primary outcomes will be described using simple descriptive statistics. Characteristics of the patients will be described as the mean (SD) or median (IQR) for continuous data and as the number of patients and percentages for categorical data. Comparison between pre and post intervention data will be analysed using Student’s *t* test or Mann-Whitney’s test for continuous data. The effect of the intervention will be estimated using 75% confidence intervals.

#### Qualitative data analysis

Focus group discussions will be audio recorded and transcribed verbatim. Data from each transcript will be analysed by two researchers initially gaining familiarisation with the texts. These will then be coded by the two researchers to identify initial themes. Once all the data has been coded and initial themes identified, there will be further discussion to determine main themes and sub-themes. Following this the original data will be revisited to consider it in relation to the themes produced. These themes will then be named and will help provide a final thematic grip [28].

### Patient and public involvement

Survivors of oesophago-gastric cancer previously treated at the Northern Oesophago-gastric Unit were approached during a cancer support group meeting. The hypothesis and design of the study was presented, and open discussion ensued; thereafter, the patients were also asked to comment on the study by completing questionnaires. They were asked to reflect on their experience in relation to the proposed study design. Themes from the discussion were recorded by one investigator (JC) and grouped into areas of agreement and comment. All the patients (100%) agreed that if they had been in the position of awaiting surgery again, they would have liked to participate in an exercise study. The additional time required attending extra tests, sessions and time needed to spend to comply with the study protocol was found to be acceptable by all respondents (100%).

In a second study six patients recovering from recent surgery for oesophageal and stomach cancer were approached on a postoperative surgical ward at the RVI, Newcastle upon Tyne. The study was introduced to them, and they were asked about their fitness levels prior to diagnosis, during chemotherapy and in the period immediately before surgery. Their opinion was sought regarding the feasibility of this study and about their willingness to participate if they had the opportunity to choose this programme prior to their surgery.

The results of the study will be shared with our oesophago-gastric cancer survivors again during cancer support group meeting once these are available.

## Discussion

It is anticipated that during an important teachable moment, the diagnosis and treatment of cancer, our patients will be open to the possibility of improving their fitness during chemotherapy and before major cancer surgery. Having previously demonstrated that fitness declines during oncological treatment we hope an exercise intervention that maintains or improves fitness could lead to better patients’ outcomes in the future. It is possible that the negative impact of NAC on cardiopulmonary fitness could be prevented by implementing a home-based prehabilitation programme during and after NAC, prior to surgery for oesophago-gastric adenocarcinoma. It is hypothesised that exploring a simple and low-cost alternative to high-impact, high-intensity, high-cost regimens could revolutionise the routine clinical care of our patients leaving them better prepared for the stress and morbidities associated with oesophago-gastric cancer surgery. This is a non-randomised, single cohort, unblinded, single-centre feasibility study with a small sample size. This design could lead to potential selection bias however the aim is to take a pragmatic approach which will allow patients to be recruited over a 1-year period and help inform a larger scale randomised control trial.

## Trial status

Recruitment to the ChemoFit started on 28.2.2019. The study is being conducted according to version 1.0 of the ChemoFit study protocol from 25 October 2018.

## Data Availability

This is the study protocol of the study which is still in progress therefore no data are available at the moment. We intend to publish our results in peer-reviewed journals and also at national and international conferences. Data will be also summarised on clinicaltrials.gov. As a requirement of our grant contract, the findings of this study will be presented to the charity funder.

## Abbreviations

NAC: Neo-adjuvant chemotherapy
NUTH: Newcastle upon Tyne Hospitals NHS Foundation Trust
CPET: Cardiopulmonary exercise testing
CT: Computed tomography
PIS: Patient information sheet
RPE: Rating of perceived exertion
GCP: Good Clinical Practice

## References

[1] Cunningham D, Allum WH, Stenning SP, Thompson JN, Van de Velde CJ, Nicolson M (2006). Perioperative chemotherapy versus surgery alone for resectable gastroesophageal cancer. N Engl J Med..

[2] Allum WH, Stenning SP, Bancewicz J, Clark PI, Langley RE (2009). Long-term results of a randomized trial of surgery with or without preoperative chemotherapy in esophageal cancer. J Clin Oncol..

[3] National Oesophago-Gastric Cancer Audit 2017 2017 [Available from: https://www.nogca.org.uk/content/uploads/2017/12/NOGCA-Annual-Report-2017.pdf.

[4] Sinclair RCF, Phillips AW, Navidi M, Griffin SM, Snowden CP (2017). Pre-operative variables including fitness associated with complications after oesophagectomy. Anaesthesia..

[5] Moran J, Wilson F, Guinan E, McCormick P, Hussey J, Moriarty J (2016). Role of cardiopulmonary exercise testing as a risk-assessment method in patients undergoing intra-abdominal surgery: a systematic review. Br J Anaesth..

[6] Jack S, West MA, Raw D, Marwood S, Ambler G, Cope TM (2014). The effect of neoadjuvant chemotherapy on physical fitness and survival in patients undergoing oesophagogastric cancer surgery. Eur J Surg Oncol..

[7] Sinclair R, Navidi M, Griffin SM, Sumpter K (2016). The impact of neoadjuvant chemotherapy on cardiopulmonary physical fitness in gastro-oesophageal adenocarcinoma. Ann R Coll Surg Engl..

[8] Khuri SF, Henderson WG, DePalma RG, Mosca C, Healey NA, Kumbhani DJ (2005). Determinants of long-term survival after major surgery and the adverse effect of postoperative complications. Ann Surg..

[9] Ulrich CM, Himbert C, Holowatyj AN, Hursting SD. Energy balance and gastrointestinal cancer: risk, interventions, outcomes and mechanisms. Nat Rev Gastroenterol Hepatol. 2018. 10.1038/s41575-018-0053-2 PMC6500387 30158569

[10] West MA, Loughney L, Barben CP, Sripadam R, Kemp GJ, Grocott MP (2014). The effects of neoadjuvant chemoradiotherapy on physical fitness and morbidity in rectal cancer surgery patients. Eur J Surg Oncol..

[11] West MA, Loughney L, Lythgoe D, Barben CP, Sripadam R, Kemp GJ (2015). Effect of prehabilitation on objectively measured physical fitness after neoadjuvant treatment in preoperative rectal cancer patients: a blinded interventional pilot study. Br J Anaesth..

[12] Blazeby JM, Conroy T, Hammerlid E, Fayers P, Sezer O, Koller M (2003). Clinical and psychometric validation of an EORTC questionnaire module, the EORTC QLQ-OES18, to assess quality of life in patients with oesophageal cancer. Eur J Cancer..

[13] Lagergren P, Fayers P, Conroy T, Stein HJ, Sezer O, Hardwick R (2007). Clinical and psychometric validation of a questionnaire module, the EORTC QLQ-OG25, to assess health-related quality of life in patients with cancer of the oesophagus, the oesophago-gastric junction and the stomach. Eur J Cancer..

[14] TNM Classification of Malignant Tumours, 8th Edition: Wiley-Blackwell; 2016.

[15] Al-Batran SE, Homann N, Pauligk C, Meiler J, Kasper S, Goetze TO (2019). Perioperative chemotherapy with fluorouracil plus leucovorin, oxaliplatin, and docetaxel versus fluorouracil or capecitabine plus cisplatin and epirubicin for locally advanced, resectable gastric or gastro-oesophageal junction adenocarcinoma (FLOT4): a randomised, phase 2/3 trial. Lancet..

[16] Weisman I (2003). ATS/ACCP Statement on Cardiopulmonary exercise testing. American Journal of Respiratory and Critical Care Medicine..

[17] Okwose NC, Avery L, O'Brien N, Cassidy S, Charman SJ, Bailey K (2019). Acceptability, Feasibility and Preliminary Evaluation of a Novel, Personalised, home-based physical activity intervention for chronic heart failure (Active-at-Home-HF): a pilot study. Sports Med Open..

[18] Physical activity guidelines: UK Chief Medical Officers' report.

[19] Borg GA (1982). Psychophysical bases of perceived exertion. Med Sci Sports Exerc..

[20] ATS/ACCP Statement on cardiopulmonary exercise testing. Am J Respir Crit Care Med. 2003;167(2):211-77. 10.1164/rccm.167.2.211 12524257

[21] Perthen JE, Ali T, McCulloch D, Navidi M, Phillips AW, Sinclair RCF (2018). Intra- and interobserver variability in skeletal muscle measurements using computed tomography images. Eur J Radiol..

[22] Sim J, Lewis M (2012). The size of a pilot study for a clinical trial should be calculated in relation to considerations of precision and efficiency. J Clin Epidemiol..

[23] Lancaster GA, Dodd S, Williamson PR (2004). Design and analysis of pilot studies: recommendations for good practice. J Eval Clin Pract..

[24] Navidi M, Phillips AW, Griffin SM, Duffield KE, Greystoke A, Sumpter K (2018). Cardiopulmonary fitness before and after neoadjuvant chemotherapy in patients with oesophagogastric cancer. Br J Surg..

[25] Gillis C, Li C, Lee L, Awasthi R, Augustin B, Gamsa A (2014). Prehabilitation versus rehabilitation: a randomized control trial in patients undergoing colorectal resection for cancer. Anesthesiology..

[26] Bousquet-Dion G, Awasthi R, Loiselle S, Minnella EM, Agnihotram RV, Bergdahl A (2018). Evaluation of supervised multimodal prehabilitation programme in cancer patients undergoing colorectal resection: a randomized control trial. Acta Oncol..

[27] Ngo-Huang A, Parker NH, Wang X, Petzel MQB, Fogelman D, Schadler KL (2017). Home-based exercise during preoperative therapy for pancreatic cancer. Langenbecks Arch Surg..

[28] Braun V, Clarke V (2006). Using thematic analysis in psychology. Qualitative Research in Psychology..

